# Supplementary material for: A Golgi Apparatus-Targeting, Naphthalimide-Based Fluorescent Molecular Probe for the Selective Sensing of Formaldehyde
Source: Molecules. 2021 Aug 17;26(16):4980. doi: 10.3390/molecules26164980 (PMC8401398; doi:10.3390/molecules26164980)
Supplement: Supplementary file 1 [file molecules-26-04980-s001.zip › molecules-1314838-supplementary.pdf]

Supporting Information

A Golgi Apparatus-targeting and Napthalimide-based Fluorescent Molecular Probe for Selective Sensing of Formaldehyde

Maxine Mambo Fortibui,[1] Wanyoung Lim,[2] Sohyun Lee,[a] Sungsu Park,[2,3]* Jinheung Kim[1]*

[1]Department of Chemistry and Nano Science, Ewha Womans University, Seoul, 03760, Korea

[2]Department of Global Biomedical Engineering, [3]School of Mechanical Engineering Sungkyunkwan University, Suwon 16419, Korea

[**]

This work was supported by the National Research Foundation (NRF) grant funded by the Korean government (NRF-2019R1A2C1007278, NRF-2017R1A5A1015365) and H-guard (2018M3A6B2057299 to S. Park) through the NRF funded by the Ministry of Science and ICT.

Experimental

Kinetic studies. The rate constant was determined from the fluorescence titration data based on a reported method [1]. The reaction of probe EW2 (5 μM) with FA at varied concentrations in PBS (10 mM, pH 7.4, 1 % DMSO) was monitored using the fluorescence intensity at 543 nm. The reaction was carried out at room temperature. The pseudo-first-order rate constant for the reaction was determined by fitting the fluorescence intensities of the samples to the pseudo-first-order equation:

$$Ln[(F_{max} - F_t) / F_{max}] = - k't$$

Where F_t and F_{max} are the fluorescence intensities at 543 nm at time t and the maximum value obtained after the reaction was completed, and k′ is the pseudo-first order rate constant. The pseudo-first-order plots for the reaction of probe EW2 (Na-FA-ER?) with 50 equiv. FA is shown in Figure S3, The negative slope of the line provides the pseudo-first-order rate constant for FA.

Determination of the fluorescence quantum yield. Fluorescence quantum yield (Φ_F) was determined by using quinine sulfate (Φ_F = 0.58, in 0.1 M H_2SO_4 aqueous solution) as the fluorescence standard. The quantum yield was calculated using the following equation [2].

$$\Phi_F(X) = \Phi_F(S) (A_S F_X / A_X F_S) (n_X/n_S)^2$$

Where Φ_F is the fluorescence quantum yield, A is the absorbance at the excitation wavelength, F is the area under the corrected emission curve, and n is the refractive index of the solvent used. Subscripts S and X refer to the standard and to the unknown, respectively.

Determination of detection limit. The linear relationship between the fluorescence intensity and the concentration of FA was fitted based on the fluorescence titration. The detection limit was calculated using the following equation based on the fluorescence titration [3].

$$\text{Detection limit} = 3\sigma/k$$

Where σ is the standard deviation of the blank sample and k is the slope of the linear regression equation.

[1] T. J. Dale, J. Rebek, Fluorescent Sensors for Organophosphorus Nerve Agent Mimics. *J. Am. Chem. Soc.* **2006**, *128*. 4500-4505.

[2] D. Oushiki, H. Kojima, T. Terai, M. Arita, K. Hanaoka, Y. Urano, T. Nagano, Development and Application of a Near-Infrared Fluorescence Probe for Oxidative Stress Based on Differential Reactivity of Linked Cyanine Dyes. *J. Am. Chem. Soc.* **2010**, *132*. 2795-2801.

[3] F. Ma, M. Liu, Z. Wang and C. Zhang, Multiplex detection of histone-modifying enzymes by total internal reflection fluorescence-based single-molecule detection. *Chem. Commun*. **2016**, *52* 1218-1221.

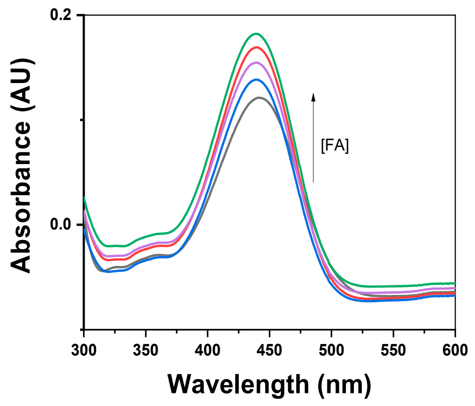

Figure S1. Absorption spectrum of the probe EW2 (10 μM) with increasing concentration of FA in PBS buffer (10 mM, pH 7.4, 1 % DMSO).

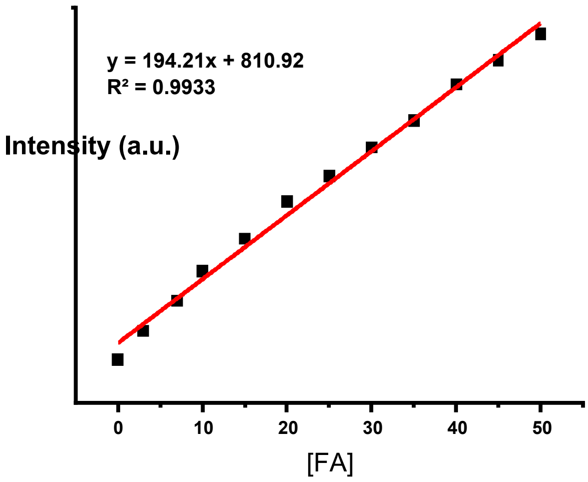

Figure S2. Linear reaction between fluorescence intensity of the probe EW2 (5 μM) and concentration of FA in PBS.

(a)

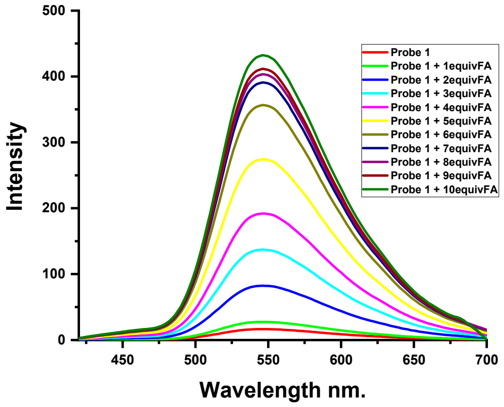

(b)

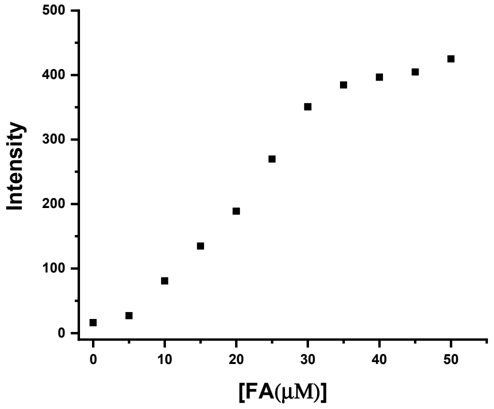

Figure S3. (a) Two photon emission of probe EW2 (10 μM) with different concentrations of FA (0 – 0.1 mM), excited at 800 nm, respectively, in 10 mM SPB buffer (pH 7.4) at room temperature. (b) Plots of emission intensity vs. concentration of FA.

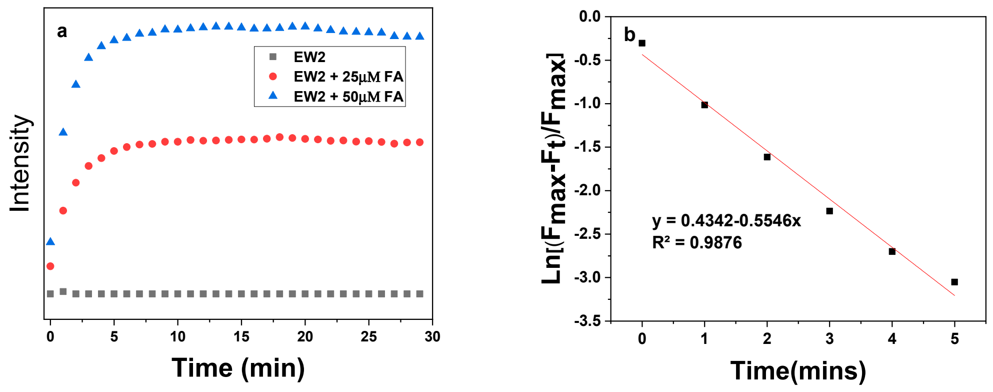

Figure S4. (a) Reaction-time profiles of probe EW2 (5 μM) in the presence of FA (0, 25, and 50 μM). (b) Pseudo-first-order kinetic plot of the reaction of EW2 (5 μM) with FA (10 equiv.) in PBS (10 mM). Slope = 0.55 min^{-1}.

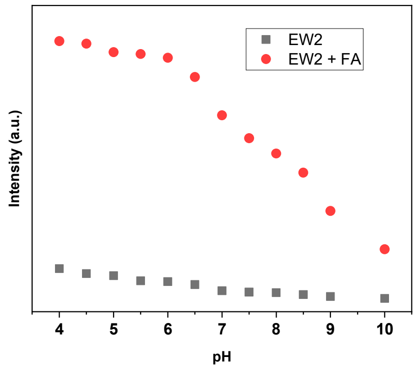

Figure S5. Fluorescence intensity changes of probe EW2 (5 μM) alone (■) and treated with 50 μM FA (●) at different pH values.

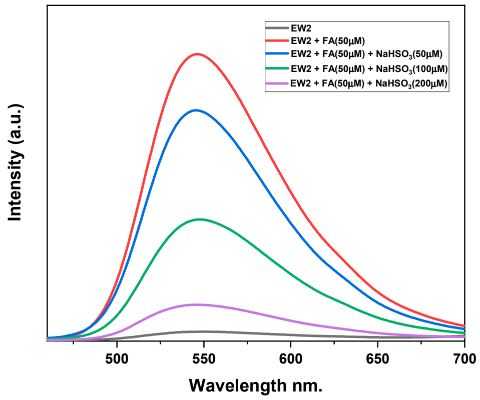

Figure S6. Emission spectra of probe EW2 (10 μM) in the absence (black) and presence (red) of formaldehyde (50 μM) in SPB buffer pH 7.3. The blue line indicates the spectrum of probe EW2 with formaldehyde and $NaHSO_3$ (50, 100, and 200 μM).

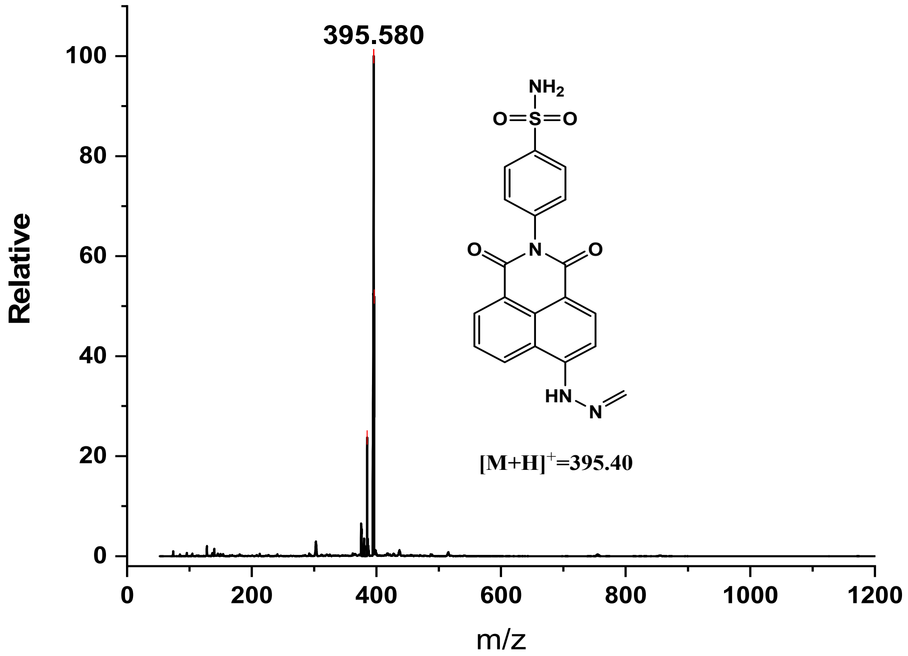

Figure S7. ESI-MS spectrum of probe EW2 treated with FA. The cation [compound **3** + H^+]$^+$ was observed at m/z = 395.58 and calculated as 395.40.

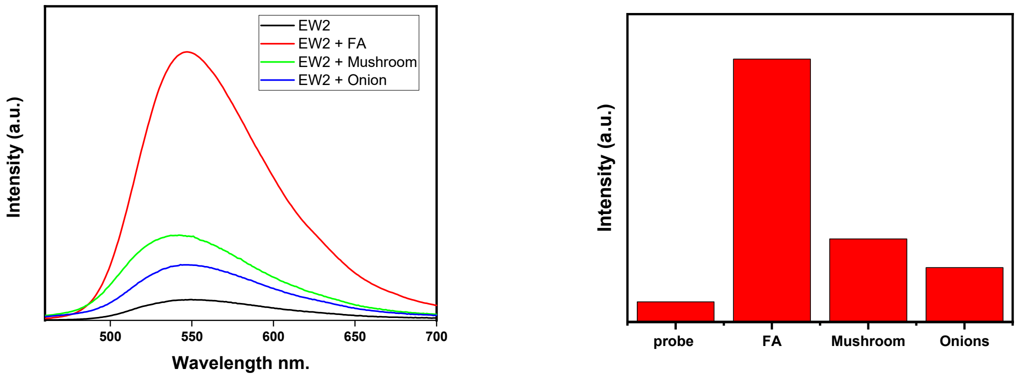

Figure S8. Emission spectra of probe EW2 (5 μM) in the presence of FA (50 μM), dried shiitake mushroom and onion extracts (each 100 μl).

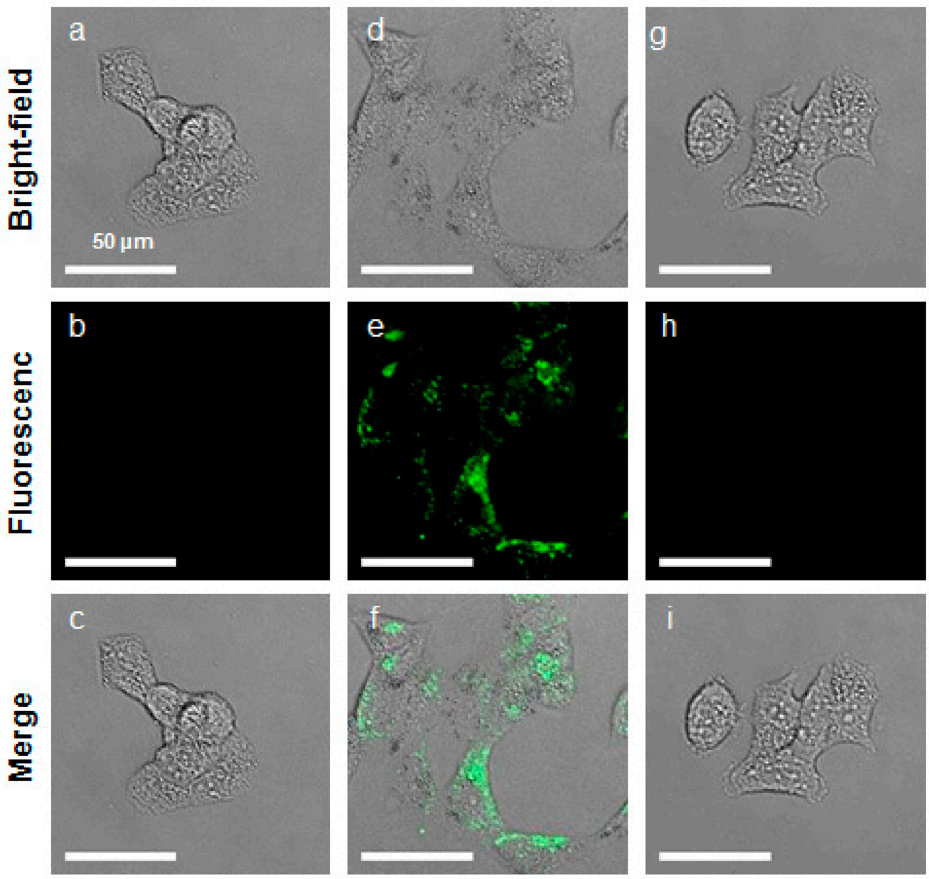

Figure S9. Fluorescence imaging of endogenous FA in the living MCF7 cells. (a) Bright-field image of live MCF7 cells treated with $NaHSO_3$ (200 μM); (b) fluorescence image of a; (c) merged image of a and b: (d) Bright-field image of live MCF7 cells treated with FA (500 μM) and EW2 (5 μM) (e) fluorescence image of d; (f) merged image of d and e: (g) Bright-field image of live MCF7 cells treated with FA (500 μM), $NaHSO_3$ (500 μM), and then with EW2 (5 μM);(h) fluorescence image of g; (i) merged image of g and h.

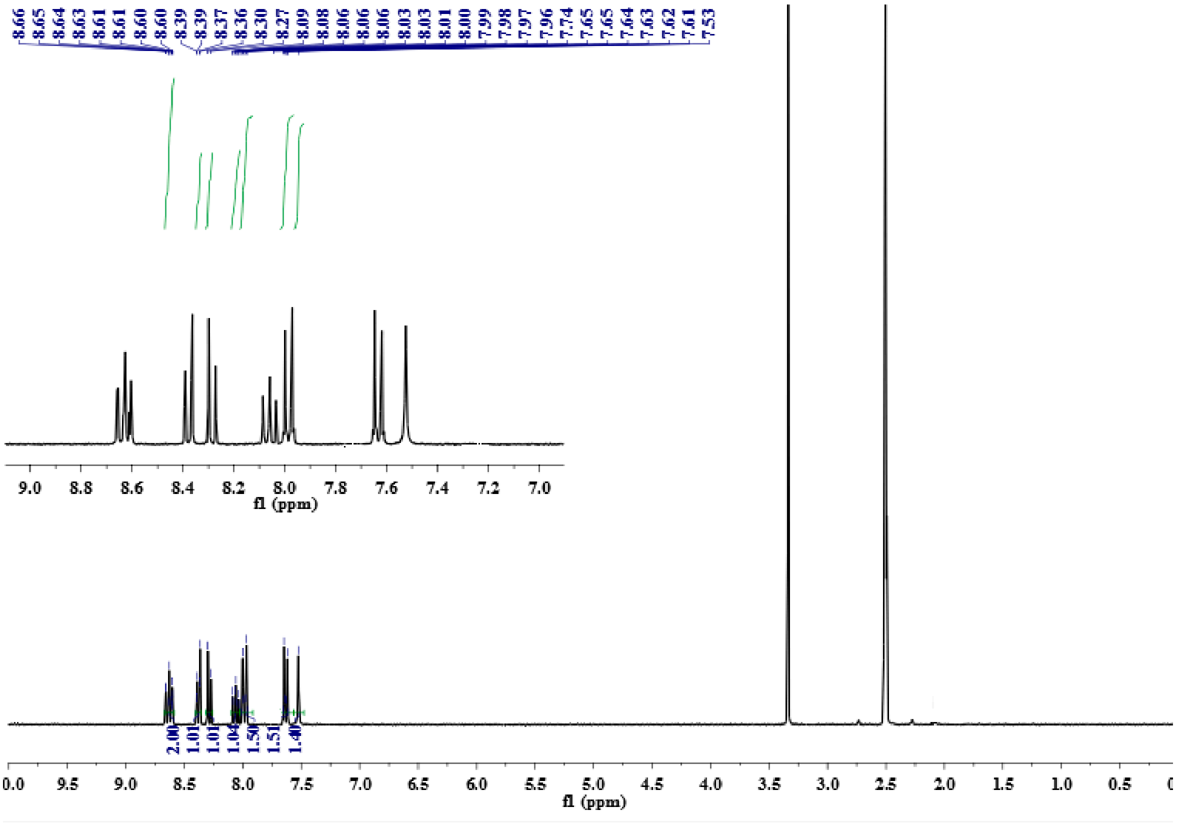

Figure S10. ^{1}H-NMR spectrum of compound **1** in DMSO-d_6.

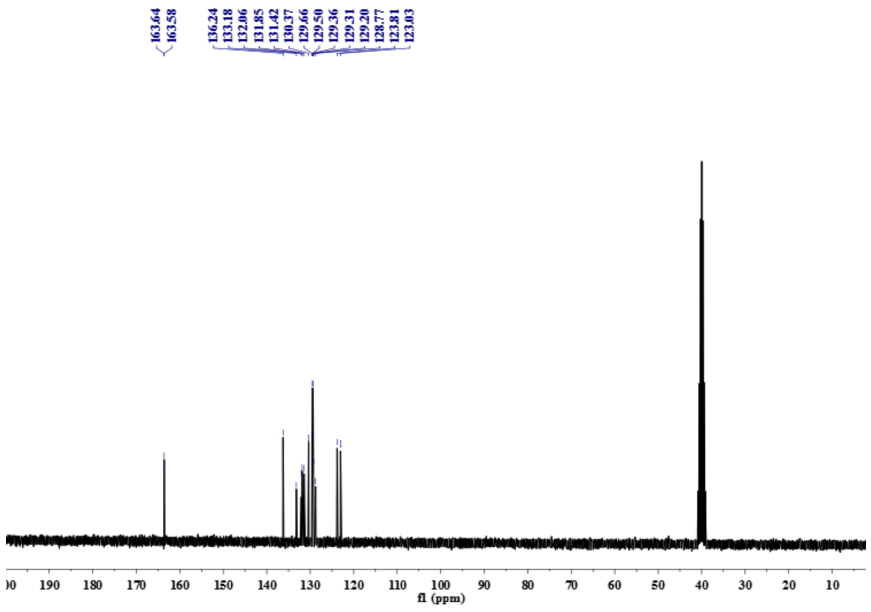

Figure S11. ^{13}C-NMR spectrum of compound **1** in DMSO-d6.

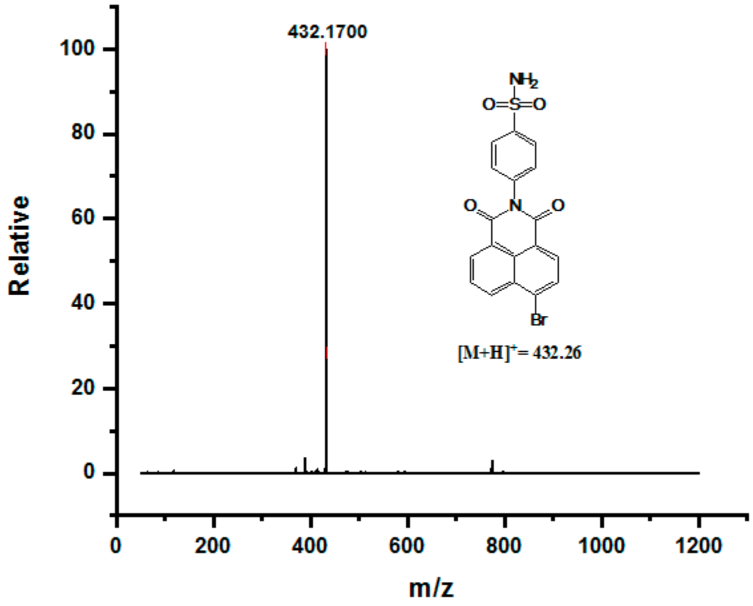

Figure S12. ESI-MS spectrum of compound **1**.

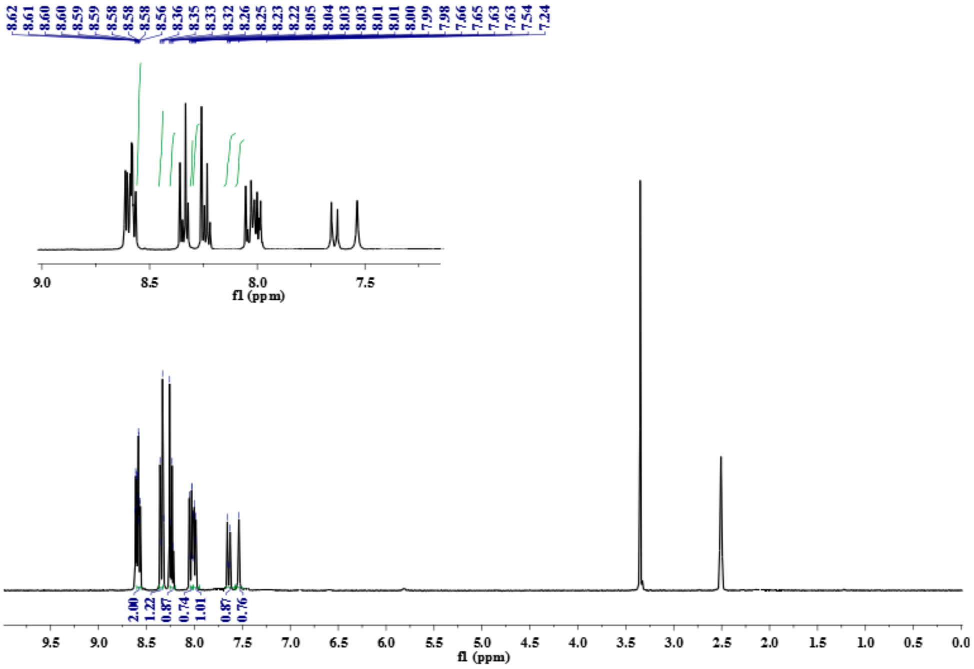

Figure S13. ^{1}H-NMR of probe EW2 in DMSO-d_6.

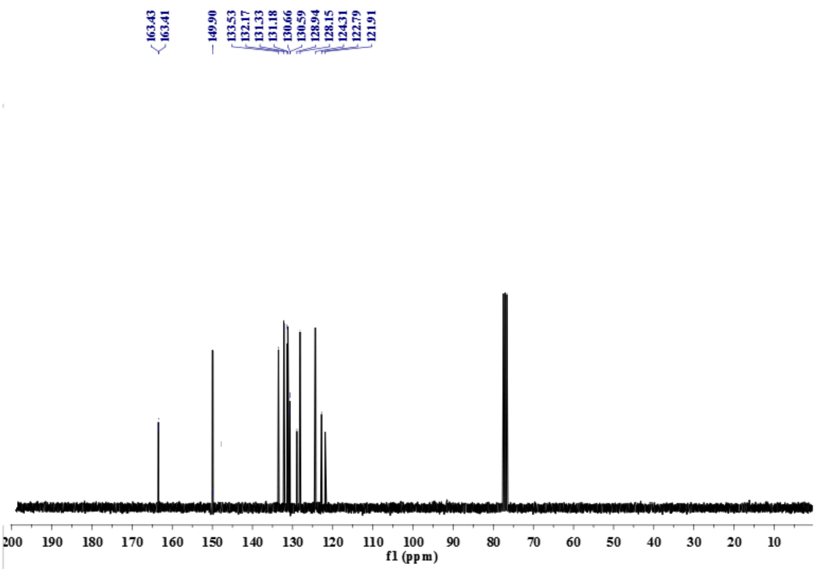

Figure S14. ^{13}C-NMR of probe EW2 in DMSO-d_6.

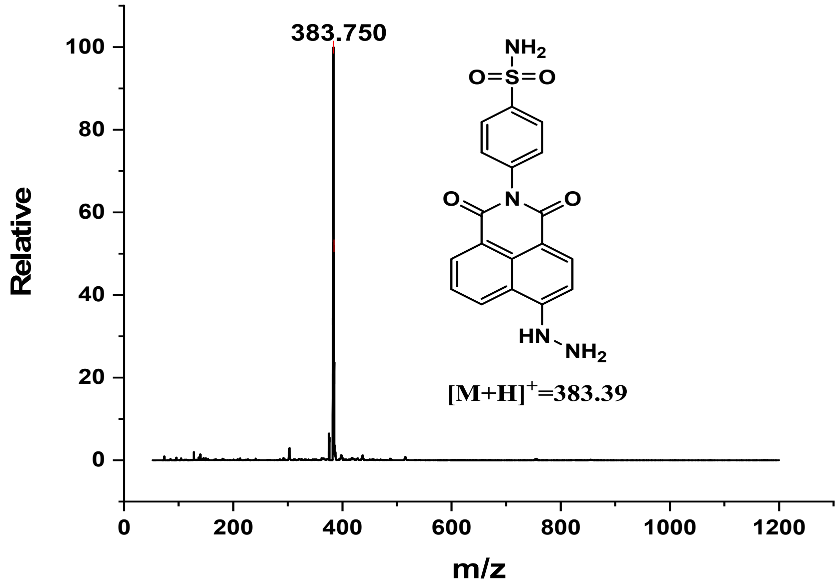

Figure S15. ESI-MS spectra of probe EW2. The cation [EW2 + H^+]$^+$ was observed at *m/z* = 383.75 and calculated as 383.39.
